# Complete response induced by fotemustine given as single agent in a patient with primary central nervous system non-Hodgkin aggressive lymphoma relapsed after high-dose chemotherapy and autologous stem cell support

**DOI:** 10.3109/10428194.2011.585672

**Published:** 2011-06-30

**Authors:** Elsa Pennese, Carolina Vergine, Rosella Matera, Michela Dargenio, Pasquale Forese, Nicola Di Renzo

**Affiliations:** Hematology and Stem Cell Transplantation Unit, ‘Vito Fazzi’ Hospital, Lecce, Italy

Despite recent therapeutic advances, primary central nervous system lymphoma (PCNSL) shows the worst prognosis among all non-Hodgkin lymphomas (NHLs), with a 5-year survival rate ranging from 4 to 40% [1,2]. Front-line therapy for patients with PCNSL is a combined chemo-radiotherapy regimen. High-dose chemotherapy with autologous stem cell support (ASCT) is still under investigation as consolidation therapy [3]. This therapeutic approach has improved the complete response rate and median survival, but the prognosis of PCNSL remains poor with a high rate of fatal relapse. There is no standardized approach for refractory or relapsed PCNSL, with reported 1-year progression-free survival (PFS) ranging from 13 to 53% and 1-year overall survival (OS) of about 50% [4-7].

Fotemustine (FTM) is a third-generation nitro-sourea with elevated lipophilic properties, which contributes to facilitate its passage through the blood-brain barrier and into malignant cells. The drug has been approved in the treatment of malignant metastatic melanoma and primary brain tumors [8]. Recently the drug has been used instead of carmustine in a novel fotemustine-based high-dose conditioning regimen (FEAM) in patients with lymphoma [9]. After a follow-up of 17 months, none of the patients with documented central nervous system involvement before ASCT showed disease progression at the level of the central nervous system, which is consistent with the active passage of FTM through the blood-brain barrier.

We report here the case of a patient with relapsing PCNSL after front-line and consolidation therapy, who received fotemustine monotherapy as salvage therapy.

A 33-year-old male patient was admitted because of signs of intracranial hypertension. Cerebral computed tomography (CT) scan and magnetic resonance (MR) revealed a tumor localized in the median deep region of the brain. Immunohistochemical analysis of the tissue (stereotactic biopsy) diagnosed diffuse large B-cell lymphoma. The staging work-up was negative for other sites of disease and the definitive diagnosis was PCNSL. Front-line therapy consisted of two courses of high-dose methotrexate (8 mg/m^2^), discontinued because of persistent mucositis (World Health Organization [WHO] grade 4), followed by three courses of L-VAMP (vincristine 1.5 mg/m^2^ IV on day 1, methotrexate 1000 mg/m^2^ IV on day 1, cytosine arabinoside 100 mg/m^2^ IV on day 1, dexamethasone 10 mg/m^2^ IV on days 1-5) combined with intrathecal (IT) lyposomal cytarabine and whole-brain radiation therapy at the total dose of 40 Gy. At the end of the front-line treatment program, complete response (CR) was documented by a negative cerebral CT scan and MR. After 3 months the patient received the FEAM regimen (fotemustine 150 mg/m^2^ on days —8 and — 7; etoposide 200 mg/m^2^ on days — 6, —5, —4, and —3; cytarabine 400 mg/m^2^ on days — 6, —5, —4, and —3; melphalan 140 mg/m^2^ on day —2) with autologous stem cell support as consolidation treatment.

**Figure 1:**
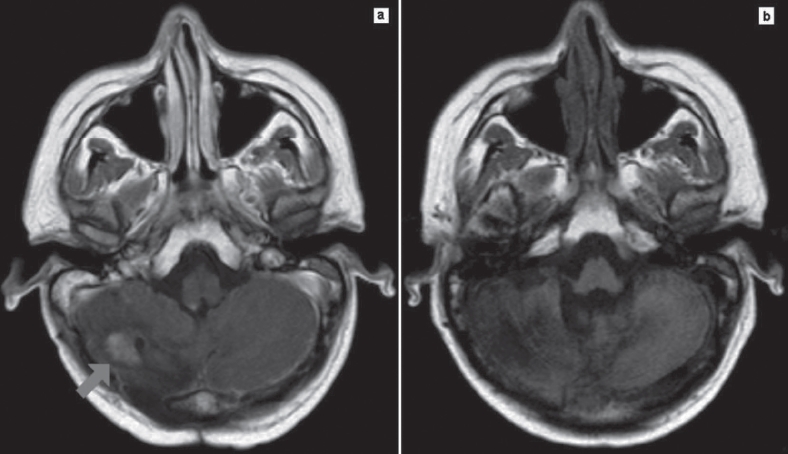
Magnetic resonance showed (a) cerebellar relapse after consolidation therapy and (b) complete response after fotemustine salvage therapy.

After a follow-up of 21 months, progressive hearing loss and ataxia appeared; CT scan and MR [Figure 1(a)] showed a cerebellar relapse. FTM was administered every 15 days at the dose of 100 mg/m^2^ as salvage therapy. The patient achieved a second complete response after eight courses of FTM, as documented by MR [Figure 1(b)].

FTM was well tolerated, with the main hematological toxicity consisting of grade II thrombocytopenia. No extrahematological toxicities were recorded. After a follow-up of 17 months the patient is still in CR, as confirmed by CT and positron emission tomography (PET) scans.

To our knowledge this represents the first report of CR achieved with fotemustine as a single agent in a patient with PCNSL relapsing after high-dose therapy and stem cell support. It is noteworthy that complete response in this patient has been achieved despite previous exposure to fotemustine, suggesting that the drug does not induce resistance.

PCNSLs are rare, aggressive malignancies with a poor outcome, but potentially curable tumors. The poor prognosis of patients with PCNSL is mainly due to the low incidence of the disease, which does not permit a consistent number of patients to be enrolled in randomized clinical trials. In addition, the advanced age of patients at diagnosis, comorbidities, and poor performance status make the diagnosis as well as optimal treatment difficult. The prognosis of relapsed-refractory patients with PCNSL is poor with the available drugs, and new agents are awaited in this field. An alkylating agent such as fotemustine could be an interesting candidate, since this drug is able to cross the blood-brain barrier. FTM is a third-generation chloroethylnitrosourea containing a phosphoalanine carrier group attached to the nitrosourea radical. The phosphoalanine group makes the drug highly lipophilic, as shown by the octanol/water partition coeffcient, which is in the optimal range compared with other nitrosoureas such as BCNU [1,3-bis(2-chloroethyl)-1-nitrosourea] and CCNU [1-(2-chloroethyl)-3-cyclohexyl-1-nitrosourea] [8]. However, more patients have to be treated and a formal trial should be conducted to define the exact role of fotemustine in the treatment of relapsing PCNSL.
